# Novel techniques for the diagnosis of neurological infections

**DOI:** 10.1097/WCO.0000000000001395

**Published:** 2025-06-05

**Authors:** Ali M Alam, Catherine F Houlihan, Tehmina Bharucha

**Affiliations:** 1 Newham University Hospital, London, U.K.; 2 University College London Hospital, London, U.K.; 3 Rare and Imported Pathogens Laboratory, United Kingdom Health Security Agency, Salisbury, U.K.; 4 Division of infection and immunity, UCL; 5 The Francis Crick Institute, London, U.K.

**Keywords:** Neurological infections, meningitis, encephalitis, myelitis, cerebrospinal fluid, diagnosis

## Abstract

**Purpose of review:**

On World Encephalitis Day 19^th^ February 2025, Encephalitis International launched the World Health Organization technical brief on encephalitis, highlighting the growing public health challenge and need for improved diagnostics. This review summarises the published literature over the last 18 months on novel methods of identifying the aetiology of neurological infections and existing research gaps.

**Recent findings:**

There is an increased availability and sensitivity of multiplex polymerase chain reaction assays and untargeted metagenomic sequencing in clinical practice. This is contributing to increasing diagnostic yield in suspected neurological infections. Preliminary results suggest that novel serological methods such as phage immunoprecipitation sequencing (Phip-seq) may be useful where molecular approaches are negative.

**Summary:**

Significant progress in improving diagnostics has been made in the last decade. Going forward, multicentre studies and meta-analyses are needed to achieve adequate power in ascertaining the role of novel diagnostic methods in neurological infections. Studies need to investigate the impact on patient management and cost-effectiveness. The role of other omics methods in identifying host biomarkers for utilisation in diagnostic algorithms needs further work.

**Search strategy:**

A database search of PubMed (MEDLINE) for English language peer-reviewed primary research articles published from 1/9/2023/9 until 28/2/2025 was conducted. Search terms used a combination of the words ‘central nervous system infection’, ‘neurological infection’, ‘cerebrospinal fluid’, ‘encephalitis’, ‘meningitis’ and ‘diagnostics’. The full search terms are reported in the Appendix.

## Introduction

Uncertainties surrounding the aetiology of patients presenting with suspected neurological infection persist in clinical practice and research. The febrile, delirious patient intubated and ventilated on intensive care, on broad spectrum antimicrobials with no diagnosis for weeks-months, if at all, is a familiar scenario. Clinicians must now deal with increasing patient complexity, particularly the amount of data to assimilate and the proportion of cases with underlying immunosuppression and a history of foreign travel, as well as dynamic epidemiology associated with urbanisation, climate change and globalisation. In tertiary care centres, we see an increasing availability of multiplex polymerase chain reaction (PCR) methods and, to a lesser extent, untargeted metagenomic next-generation sequencing (mNGS). However, it is not clear how these alter patient management. In this article, we query what progress has been made in laboratory diagnostics for community-acquired neurological infections in the last 18 months and whether there is any evidence of impact on patient outcomes.

## Pathogen-based approaches

### i Multiplex polymerase chain reaction (PCR) assays

Multiplex PCR assays target multiple pathogens in similar sample volumes as those used for conventional singleplex PCR assays, thus sparing precious cerebrospinal fluid (CSF). Several commercial assays are currently used in clinical practice for the diagnosis of central nervous system (CNS) infections. FilmArray® Meningitis/Encephalitis panel (FAME-p) (Bio-Fire Diagnostics, Biomérieux Company, UT, USA) is the most widely cited. FAME-p incorporates automated nucleic acid extraction, reverse transcription and amplification to simultaneously detect 14 pathogens in under an hour (1). Two previous meta-analyses suggest that FAME-p has sensitivity over 64.9% and specificity over 97.4%, with considerable uncertainty in these estimates (1). There are particular concerns raised about multiplex PCR entirely replacing specific singleplex assays (such as Herpes simplex virus (HSV) PCR assays) due to the risk of false negatives as well as the risk of false positive results, particularly for Streptococcus pneumoniae (1, 2). A retrospective analysis of 7,551 FAME-p results at the University of Kentucky 2016-2022 assessed its accuracy by comparing positive bacterial and fungal detections with patient CSF and blood cultures, clinical presentations, and other laboratory findings (3)**. In 29 (27.4%) of 106 patients with positive FAME-p results (excluding 9 false positives) no pathogen was identified using standard bacterial and fungal methods, highlighting its role in the rapid diagnosis of meningitis and encephalitis, particularly in patients who have received antibiotics. However, there were false positive results consistent with previous literature (1). Notably, the study did not utilise the standard method of diagnosing Cryptococcus, Cryptococcal antigen test (CrAg). Other multiplex PCR panels, such as the QIAstat-Dx® Meningitis/Encephalitis Panel (QIAGEN, Germany), have shown similar performance to FAME-p (4), (5)**, (2)**, however more data are needed.

There are additional caveats to the performance of multiplex PCR assays that may not be adequately assessed in diagnostic accuracy studies. For example, the performance of multiplex PCR is dependent on the pathogens included for detection in the panels (6, 7)*(8). There is a need to contextualise negative results with local clinical and epidemiological data and consider additional targeted PCR assays or alternative methods (e.g. CrAg for Cryptococcus, serology for Flaviviruses). Furthermore, certain diagnoses which have low prevalence and questionable clinical significance outside specific sub-populations, for example HHV6, HHV7, EBV and CMV, should rely on clinical interpretation and subsequent testing to determine clinical significance (9, 10).

There is preliminary data to suggest that multiplex PCR assay improve patient outcomes, including shortening intensive care admissions and supporting antimicrobial stewardship (11). A paediatric observational study across two UK hospitals, comparing clinical data from children with meningitis following FAME-p implementation and a historical cohort (n=460), found that FAME-p was associated with shorter courses of antibiotics and shorter hospital stays (12)**. However, these findings need to be corroborated with larger studies that also take into account the aforementioned caveats in the study design.

### ii Metagenomic next-generation sequencing (mNGS)

**Figure 1:**
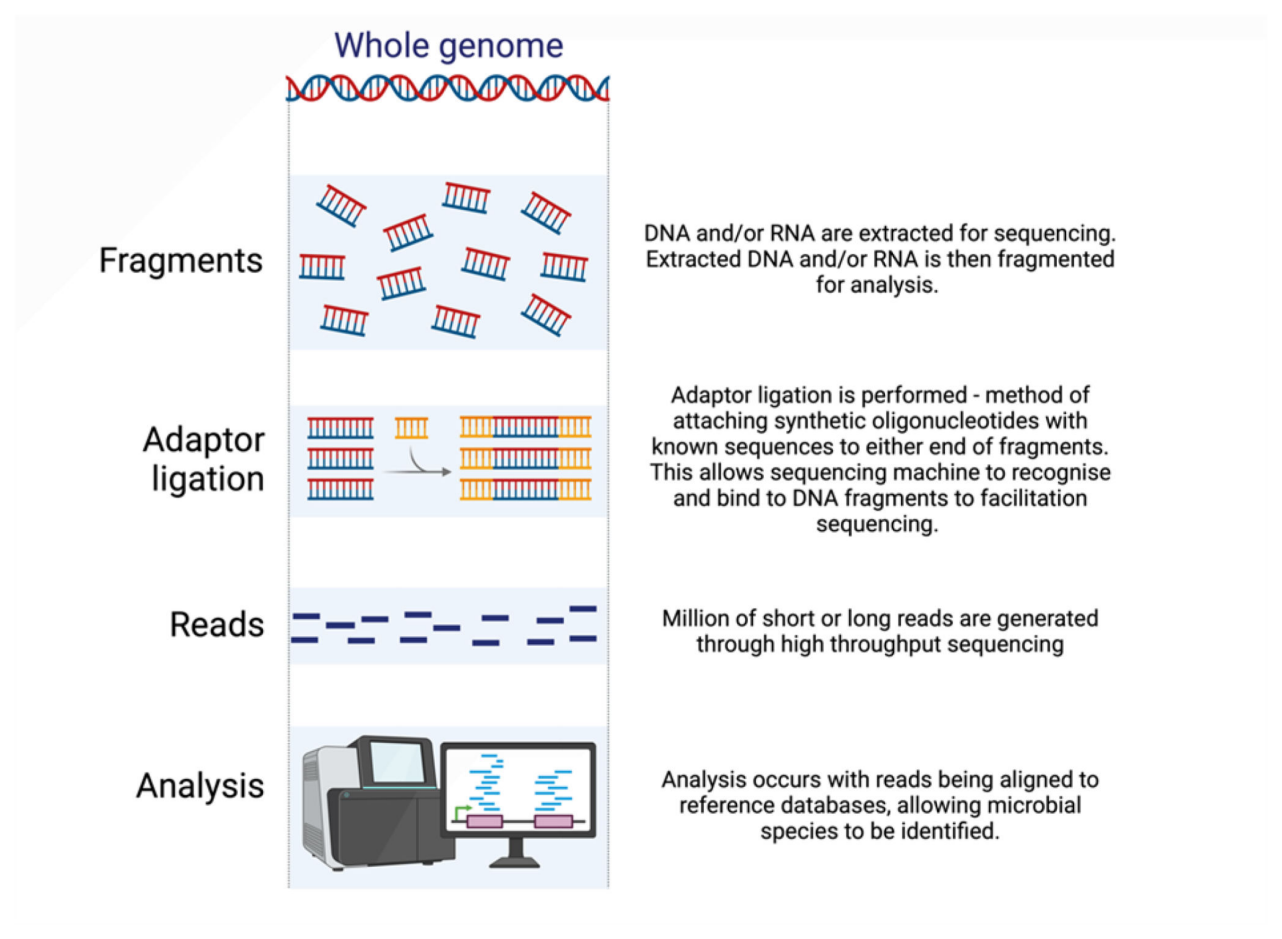
Schematic of metagenomic next-generation sequencing (mNGS). All the nucleic acid (including DNA and/or RNA) of a specimen is sequenced in parallel, allowing characterisation of microbiological genetic material. This allows identification of rare or unexpected pathogens present in a sample, which are often missed by conventional diagnostic methods. Created in BioRender. Alam, A. (2025) https://BioRender.com/i63v402

mNGS, illustrated in Figure 1, has become more widely used in research practice, and clinically in specialised tertiary care centres as an end-of-the-line diagnostic tool particularly in critically ill patients. The utility of mNGS was recently highlighted in a landmark seven-year retrospective study which analysed the performance of mNGS in almost 5,000 CSF samples (13)**. This study found that mNGS testing was the most sensitive test for the detection of pathogens in a cohort of patients with clinically severe and diagnostically challenging CNS infections, and was the only or first test to make the diagnosis in 67 (30.4%) of 220 infections. Among the 220 confirmed infectious diagnoses, mNGS alone identified 48 (21.8%) cases, highlighting its potential as an adjunct to traditional diagnostic methods. The study reported a sensitivity of 63.1% (rising to 86% when only considering CSF-based detection) and a specificity of 99.6%. Of note, mNGS exhibited significantly higher sensitivity compared to indirect serologic testing and direct detection testing from CSF and other body fluids. These findings are replicated in paediatric populations, with a meta-analysis of 10 papers demonstrating the high diagnostic performance of mNGS for CNS infections through a pooled sensitivity of 76% and specificity of 94% (14)*. The diagnostic utility of mNGS in CNS infection is also seen in brain abscesses: where mNGS has exhibited superior diagnostic performance compared to traditional culture and PCR-based methods, with the added capability of detecting a broader range of pathogens (15)*. Earlier pathogen detection through use of mNGS has been shown to positively influence patient management and antibiotic stewardship (16).

Despite its advantages, mNGS is not routinely used in clinical diagnostics due to concerns regarding its accuracy, lack of accreditation, high cost and turnaround time. It remains more expensive than conventional methods such as PCR and multiplex panels, and its workflow (from sample processing to sequencing and data analysis) can take several days to weeks. This limits its use in urgent clinical decision-making. In such settings, targeted PCR may deliver results within hours. However, mNGS can play a more specific diagnostic role in the setting of detecting unculturable, difficult-to-diagnose organisms, as well as identifying rare, unexpected infections. For example, mNGS has been instrumental in identifying unexpected pathogens. A study in southern Spain which employed mNGS of CSF in cases of aseptic meningitis with no aetiology identified Toscana virus in 10% of patients, making it among the most frequently identified pathogen, alongside enteroviruses, herpes simplex virus, and varicella-zoster virus (17)*. Furthermore, a study employing mNGS in 47 paediatric patients with suspected viral meningoencephalitis demonstrated the identification of uncommon viruses such as rotavirus, Sindbis virus and influenza A virus in CSF of cases that remained undiagnosed with routine viral panels (18)*. Another single-centre study analysing shotgun sequencing in paediatric CNS infections of unknown aetiology reported significant viral detection in 30 of 40 cases previously negative by p-FAME (19)*. This included clinically relevant pathogens such as parechovirus A, enteroviruses, HSV-1, Varicella zoster virus (VZV) and influenza A virus, as well as unexpected findings like polyomavirus 5 and HERV-K113. Notably, confirmatory PCR testing confirmed only a subset of these detections. The ability to detect a broader spectrum of potential pathogens highlights the challenges associated with mNGS, including the risk of contamination (e.g., detection of papillomavirus) and uncertainty regarding the clinical relevance of certain findings, such as the detection of endogenous retroviruses like HERV-K113.

Often, these unexpected pathogens may be present in the context of immunocompromised individuals, wherein its presence may be clinically relevant. It is in this subset of patients that may benefit greatly from use of mNGS. In people living with HIV, mNGS demonstrates a higher pathogen detection rate than in immunocompetent patients, with a greater frequency of co-infections and a higher prevalence of fungal and DNA viral pathogens (20)*. A case report in the USA on two fatal cases of meningoencephalitis in immunocompromised hosts (both transplant recipients) identified Potosi virus and Lone Star virus, bunyaviruses associated with the Lone Star tick (*Amblyomma americanum*) and prevalent in the United States (21)*. Most bunyavirus infections are asymptomatic or manifest as a mild, self-limited febrile illness; however, severe complications can occur in those immunocompromised, and the detection of bunyaviridae in these two fatal cases highlights how mNGS can identify rare infections which can carry significant consequences in vulnerable populations. This is also pertinent in public health investigations of outbreaks, as shown in the fungal meningitis outbreak linked to epidural anaesthesia in Matamoros, Mexico in 2023. mNGS and pan-fungal PCR identified Fusarium solani species complex in CSF, facilitating early recognition of the outbreak and guiding antifungal treatment strategies (22)*.

### iii Pathogen-based protein and metabolite biomarkers “antigens”

The most established “antigen” based diagnostic in current clinical practice being the CrAg, now considered the gold-standard for detection of Cryptococcal CNS infection (23). These may be detected in various formats, typically enzyme-linked immunosorbent assays (ELISA) and immunochromatographic assays ‘lateral flow tests’ (LFTs), the latter being particularly important in diagnosing patients in areas with limited access to laboratory diagnostics with resource-constraints. LFTs provide point-of-care testing, potentially at the patient’s bedside, with results available within minutes.

A previous meta-analysis has demonstrated high diagnostic accuracy (100% sensitivity and 97% specificity) of existing Streptococcus pneumoniae LFTs for CSF (24). More recently, a meta-analysis of Neisseria meningitidis LFTs demonstrated pooled sensitivity of >91% and pooled specificity >93% (25)*. In theory, the very high sensitivity of S. pneumoniae LFTs support the use of the tests in ‘ruling-out’ and potentially de-escalating unnecessary dexamethasone therapy. However, clear evidence of impact on patient management is lacking for both S. *pneumoniae* and N. *meningitidis* LFTs in CSF, and taking into account the devastating consequences of these infections, the standard diagnostic protocol where laboratory capacity is available is gram-stain, culture, antibiotic resistance testing +/- PCR. Furthermore, in contrast to the widely available S. *pneumoniae* LFTs (e.g. BinaxNow, Abbott), the only N. *meningitidis* LFT that covers all the serogroups (Meningospeed, Biospeedia) is not yet available for purchase at present.

Broad-range antigens have also been targeted in LFTs, such as Beta-D-glucan (BDG) and Galactomannan for fungal infections. A retrospective multi-centre study in France suggests that BDG testing in CSF demonstrates suboptimal diagnostic accuracy for non-cryptococcal fungal infections (26)*. A meta-analysis of the use of galactomannan for Aspergillus demonstrates poor sensitivity (69.0% 95% CI, 57.2-78.7%) but reasonable specificity (94.4% 95% CI, 82.8-98.3%) (27)*.

## Host response approaches

Conventional laboratory methods incorporating the host response involve detection of antibodies in the intrathecal space (ITAb), discussed below. An alternative discipline is the harnessing of host biomarkers such as C-reactive protein or Interleukin 6 (IL-6) to discriminate between groups of patients (e.g. CNS infections vs. other diseases and bacterial vs. viral infections) or to identify specific aetiologies. There is emerging application of large-scale study ‘omics’ methods, namely transcriptomics detecting ribonucleic acid (RNA), proteomics detecting proteins (including antibodies) and metabolomics detecting metabolites (including glycans and lipids), in harnessing novel host response biomarkers (28). Overall, these methods remain in the research arena rather than routine clinical practice, as they have frequently demonstrated suboptimal specificity (29). However, improving accessibility, cost and turnaround time of these methods has been associated with incremental progress in these domains. Current strategies involve untargeted approaches combined with predictive modelling to select host molecular signatures, rather than aiming to identify upregulation of a single biomarker, and may be combined with pathogen-based markers to achieve clinically useful diagnostic metrics (30).

### i Serology

The detection of pathogen-specific ITAb production has supported the diagnosis of CNS infection when molecular methods have failed. The test is most commonly used for spirochetes (*Borrelia burgdorferi* and *Treponema pallidum* for neuro-lyme and neuro-syphilis respectively), neurotropic herpesviruses (HSV1/2 and VZV), flaviviruses (Tick-borne encephalitis, West Nile virus, Japanese encephalitis etc), and specific conditions such as subacute sclerosing panencephalitis (SSPE) caused by measles. The technique for most, but not all, of these requires an age-based calculation which determines whether there is a higher concentration of the specific antibody in the intrathecal space compared to serum (31). The turn-around time for most ITAb tests are a month, and so are useful mostly in retrospective diagnosis or chronic undiagnosed conditions.

The most commonly used (and CE marked, therefore being deemed to meet EU safety, health and environmental protection requirements) assays for ITAbs are ELISAs. Chemiluminescent assays have been developed, which may offer a wider dynamic range and, in direct comparison with ELISA for HSV1/2 and VZV, have demonstrated improved sensitivity and specificity (32). Most ITAb tests use IgG since the incubation period before neurological presentation is sufficient to allow an IgM to IgG class switch. In tick-borne encephalitis (TBE), similar to other flaviviruses, the presence of intrathecal anti-TBE IgM is used in diagnosis (33).

Attempts have been made to compare characteristics, including outcome, of patients with PCR-confirmed HSV /VZV encephalitis with patients with only raised ITAb indices for these viruses. However, patients with evidence of ITAb production appear to receive a diagnosis of CNS infection in less than 50% of cases, severely limiting any interpretation of this comparison (34).

The use of a quantitative IgG in the CSF and serum has been used to diagnose Mpox encephalitis. Although experimental, it appears valid when used in the context of PCR-confirmed Mpox in skin lesions with contemporaneous neurological symptoms (and PCR-negative CSF). Animal model experiments have been used to explore whether IgG and IgE production in the intrathecal space are useful in the diagnosis of parasitic infections such as *Angiostrongylus cantonensis*, which infects the CNS in humans and is challenging to diagnose (35).

Developments in serological assays used in blood have been applied to CNS infections. Phage immunoprecipitation sequencing (PhIP-Seq) is a high throughput method for profiling antibody responses by utilising bacteriophages to display a library of millions of peptides derived from pathogens. Profiling antibodies in this way has revealed unexpected diagnosis such as chronic encephalitis from dengue several years ago (36). More recently, the utility of Phip-Seq has been demonstrated by identifying enterovirus D68, a common neurotropic virus, as the cause of Guillain-Barré syndrome in a child (37). It is expected that this emerging technique will provide advancement in diagnostics for CNS infection in combination with other methods in this paper.

### ii Transcriptomics

Similar laboratory methods for analysing pathogen genomes may be utilised for exploring host gene expression (host RNA), transcriptomics, in response to an infectious pathogen. For this reason, transcriptomics data is frequently a by-product of mNGS and represents accessible data for investigation. Tuberculous meningitis is notoriously difficult to diagnose using pathogen-based approaches, and the application of host-based methods has been an active field of research, including incorporating host RNA signatures (30). For pulmonary tuberculosis, a commercial point-of-care assay ‘Xpert MTB Host Response’ has been developed, based on PCR detection of 4 host transcripts in blood. A recent publication evaluated the performance of the same genes for discriminating TB meningitis (TBM) from pulmonary TB, as well as from bacterial and viral meningitis (38)**. One of the three genes included in the panel, guanylate-binding protein 5 (GBP5), measured in whole blood discriminated TBM from other brain infections (area under the curve (AUC) 0.74; 95% confidence interval [CI], 0.67–0.81) as well as in individuals with HIV (AUC, 0.86; 95% CI, 0.80–0.93). This work represents a significant advance in the field of host-based diagnostics. However further verification of the findings is required, as well as evaluation of the diagnostic yield when measured together with pathogen-based methods.

### iii Proteomics and metabolomics

Novel biomarker discovery using proteomics and metabolomics represents more nascent disciplines compared to transcriptomics. The pipeline for untargeted analysis takes years, conventionally relying on mass spectrometry methods followed by laborious antibody-based confirmation. Studies are frequently performed on remnants of CSF and are underpowered such that they do not proceed beyond early discovery analysis; accurate predictions based on host biomarkers require relatively large (100-1,000 patient) datasets (39). For proteomics, some of these issues are being circumvented by high-throughput large targeted multiplex platforms such as Olink and Somascan, however these are currently prohibitively expensive.

For the aforesaid reasons, proteomics studies frequently utilise targeted assays such as Luminex panels to detect a small number of known targets, for example 1-20 analytes. This is exemplified by a systematic review summarising the literature specifically for paediatric bacterial meningitis which reported pooled meta-analyses accuracy metrics for the best studied biomarkers, CRP, procalcitonin, IL6, IL8, TNF-alpha and ferritin. These data suggest that testing the biomarkers in CSF, but not blood, provide good discrimination of bacterial vs viral meningitis as well as infectious vs non infective aetiologies (40)**. The authors acknowledge that these data are largely based on low quality heterogeneous studies testing samples retrospectively, and that none of the biomarkers demonstrate improved accuracy in detecting bacterial meningitis as compared to established tests such as CSF leucocyte count. These findings are supported by a subsequent study in 61 neonates utilising a Luminex panel detecting 12 cytokines, chemokines, and acute phase reactants (CRP, procalcitonin, CXCL-10, MDC, IL6, Il-8, IL-10, TNF-a, MIF, IL-1RA, CXCL13, IL-1B) in blood and CSF to differentiate meningitis and sepsis (41)**. The best discriminating protein was IL6 (Area under the curve [AUC] of 0.94 (95% CI 0.85-1.00), however this was superseded by CSF leucocyte count alone with an AUC 0.96 (95% CI 0.89-1.00). In adults, the same group reported using the Luminex panel in a cohort of 738 patients CSF samples to identify all CNS infections (42). Again, CSF leucocyte count outperformed the protein biomarkers in differentiating CNS infections from other diseases, and if CNS leucocyte count >1,000/mm^3^ also for differentiating bacterial meningitis from other diseases, and bacterial and viral CNS infections. However, in the subgroup of patients with a CSF leucocyte count 5-1,000/mm^3^, other biomarkers such as a combination of CRP and IL6 demonstrated better predictive accuracy in differentiating bacterial meningitis from other diseases and bacterial vs viral CNS infections. In line with previous literature suggesting that proteins associated with neutrophil activation (e.g. Myeloperoxidase, Lactotransferrin, S100 A8/9 (Calprotectin), S100B and Lipocalin 2) have high predictive accuracy in diagnosing bacterial meningitis, a systematic review and meta-analysis suggest that heparin binding protein (azurocidin) has high diagnostic accuracy for bacterial meningitis (pooled sensitivity and specificity ≥95%), (43)*. In contrast, a systematic review and meta-analysis was performed specifically for the role of CSF and serum procalcitonin in identifying CNS infections, with sensitivity and specificity below 90% (44)*. It remains to be determined if heparin-binding protein outperforms the conventional biomarker neutrophil%, and how it might perform together with other novel protein biomarkers. In terms of specific novel biomarkers, another study measured the extracellular matrix glycoprotein, Tenascin-C, in 174 patient CSF samples and demonstrated it to be significantly higher in bacterial meningitis vs other CNS infections or non-infectious patients (45)*. The study did not report accuracy metrics, and the work needs verification in a different cohort of patients.

Beyond bacterial meningitis, researchers have analysed diagnostic biomarkers of specific neurological infections. A study of CSF samples from patients with laboratory confirmed enterovirus meningitis (n=38) vs age and gender-matched non-infectious controls (n=19) (46)*. There were no increased levels of GFAP or NFL in enterovirus meningitis, however there were decreased levels of Aβ42, Aβ40, T-tau, P-tau and S-100B. A study quantified NFL in 20 individuals with uncomplicated syphilis, 10 with asymptomatic neurosyphilis and 10 with symptomatic neurosyphilis; CSF NFL was significantly higher in those patients with symptomatic neurosyphilis (47).

Application of metabolomics is less well explored. A study reports preliminary explorative analyses of the application of metabolomics to differentiating infectious and autoimmune encephalitis, however verification in external cohorts is required (48). A meta-analysis of lipoarabinomannan in CSF for diagnosing TBM suggests that this does not exhibit sufficient diagnostic accuracy for use (49).

## Research gaps

Achieving adequate power is a key challenge of this research; suspected cases are infrequent, CSF is an invasive sample that is not easy to obtain in sufficient quantity and aetiologies are heterogeneous. For these reasons, collaboration between researchers in multi-centred studies and meta-analyses is crucial. It is noteworthy that much of the research described needs to be replicated to inform clinical practice. Moreso, there is a sparse data on patient impact of these diagnostics including mortality and cost-effectiveness. For metagenomic sequencing, further work is needed on developing pathways and consensus for interpretating and validating mNGS results. Finally, once identified, novel diagnostics must be translated into near-patient low-cost test, stable enough for use in low-middle income settings.

## Conclusion

Neurological infections are a growing public health challenge and there is a need for improved diagnostics. There is an increased availability of multiplex polymerase chain reaction assays, untargeted metagenomic sequencing and novel serological methods in clinical practice, which is contributing to increasing diagnostic yield in suspected neurological infections. However, these methods have drawbacks; multicentre studies and meta-analyses are needed to achieve adequate power in ascertaining the role of novel diagnostic methods in neurological infections.

## Supplementary Material

Appendix

## Key points

- Multiplex polymerase chain reaction (PCR) assays allow rapid and simultaneous detection of pathogens but these need to be carefully validated.
- Metagenomic next generation sequencing is increasingly used for complex cases.Lateral flow tests using antigen-based diagnostics are important in resource poor settings.Serological tests such as phage immunoprecipitation sequencing (harness host response biomarkers to aid diagnosis and show potential.
