# Supplementary material for: Novel techniques for the diagnosis of neurological infections
Source: Curr Opin Neurol. Author manuscript; Available in PMC 2025 Nov 13. (PMC7618343; doi:10.1097/WCO.0000000000001395)
Supplement: Appendix [file EMS210349-supplement-Appendix.docx]

## Methods

PubMed search 12/02/2025: (("cerebrospinal fluid"[Title/Abstract] OR "CSF"[Title/Abstract] OR "neurological infectio*"[Title/Abstract] OR "central nervous system infectio*"[Title/Abstract] OR "cns infectio*"[Title/Abstract] OR "meningit*"[Title/Abstract] OR "encephalit*"[Title/Abstract]) AND "diagnos*"[Title/Abstract]) AND (2023:2025[pdat]) Filters: from 2023/9/1 - 2025/2/28

Figure 1: PRISMA flow diagram


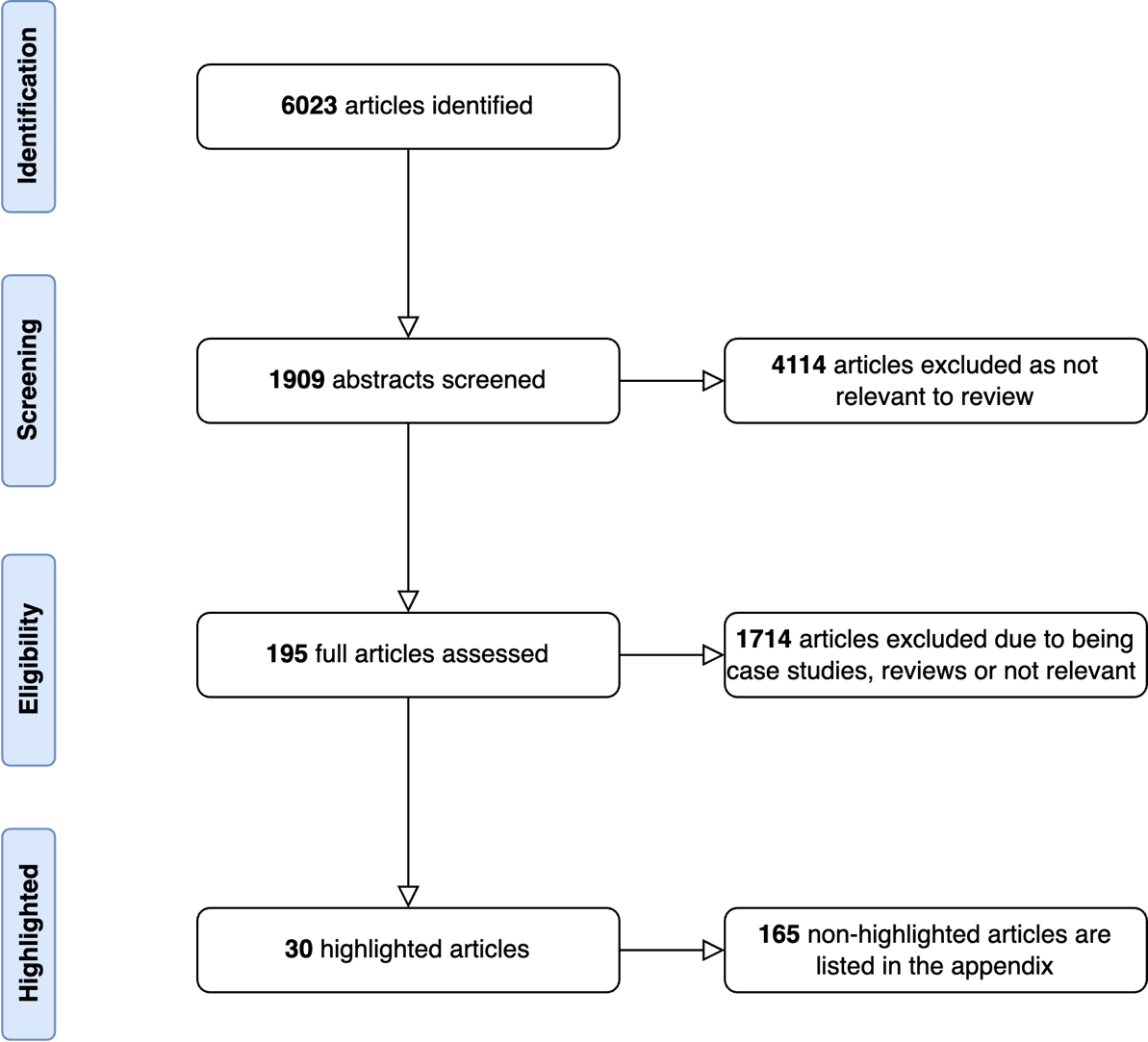


## Results

References of special interest are marked * and those of outstanding interest are marked **.

### Polymerase chain reaction methods (PCR)

1. **Cuesta G, Puerta-Alcalde P, Vergara A, Roses E, Bosch J, Casals-Pascual C, et al. An Assessment of a New Rapid Multiplex PCR Assay for the Diagnosis of Meningoencephalitis. Diagnostics (Basel). 2024;14(8).
2. **Myint T, Soria J, Gao Y, Conejo Castillo MR, Arora V, Ribes JA. Comparison of positive BioFire FilmArray meningitis/encephalitis (ME) panels, CSF cultures, CSF parameters, clinical presentation and in-patient mortality among patients with bacterial and fungal meningitis. *Microbiol Spectr.* 2025 Feb 4;13(2):e0001424. doi: 10.1128/spectrum.00014-24. Epub 2024 Dec 23. PMID: 39714177; PMCID: PMC11792450.
3. ** Sundelin T, Bialas J, de Diego J, Hermanowski M, Leibhan H, Ponderand L, Juanola-Falgarona M, Jones T, Rey M, Johnson S, Pareja J, Caspar Y. Evaluation of the QIAstat-Dx Meningitis/Encephalitis Panel, a multiplex PCR platform for the detection of community-acquired meningoencephalitis. *J Clin Microbiol.* 2023 Oct 24;61(10):e0042623. doi: 10.1128/jcm.00426-23. Epub 2023 Sep 13. PMID: 37702495; PMCID: PMC10595057.
4. **Sunnerhagen T, Widén J, Handhal S, Özkaya Şahin G. A retrospective observational study of 1000 consecutive patients tested with the FilmArray® Meningitis/Encephalitis panel: clinical diagnosis at discharge and microbiological findings. Scientific reports. 2024;14(1):4015.
5. **Kadambari S, Feng S, Liu X, Andersson M, Waterfield R, Fodder H, et al. Evaluating the Impact of the BioFire FilmArray in Childhood Meningitis: An Observational Cohort Study. Pediatr Infect Dis J. 2024;43(4):345-9.
6. Humisto A, Antikainen J, Holma T, Jarva H, Toivonen A, Loginov R, et al. Evaluation of the Novel CE-IVD-Marked Multiplex PCR QIAstat-Dx Meningitis/Encephalitis Panel. Microbiology spectrum. 2023;11(3):e0514422.
7. Dong DV, Boutin S, Sang VV, Manh ND, Hoan NX, Quang HX, Lien TT, Trang VD, The NT, Linh LTK, Schmauder K, Ueltzhöffer V, Hafza N, Hauswaldt S, Rupp J, Kremsner PG, Song LH, Nurjadi D, Peter S, Velavan TP. Optimization of the Diagnosis of Central Nervous System Infections in Vietnamese Hospitals: Results From a Retrospective Multicenter Study. *Open Forum Infect Dis.*2024 Sep 13;11(9):ofae531. doi: 10.1093/ofid/ofae531. PMID: 39346707; PMCID: PMC11429109.
8. Kitagawa D, Kitano T, Uchihara Y, Ando T, Nishikawa H, Suzuki R, et al. Impact of Multiplex Polymerase Chain Reaction Test in Patients With Meningitis or Encephalitis. Open Forum Infect Dis. 2023;10(12):ofad634.
9. Pathak A, Pham C, Shay S, Lasco T, Al Mohajer M. The impact of the FilmArray meningitis/encephalitis panel on empiric antibiotic prescriptions in patients with suspected community-acquired meningitis. Antimicrob Steward Healthc Epidemiol. 2024;4(1):e104.
10. Ben Lahlou Y, Eddair Y, Dokponou YCH, Elouennass M, Chadli M. The Evaluation of the BioFire FilmArray Meningitis/Encephalitis Panel for the Detection of Bacteria and Yeast in Cerebrospinal Fluid Specimens. *Cureus.* 2024 Mar 16;16(3):e56260. doi: 10.7759/cureus.56260. PMID: 38623118; PMCID: PMC11017369.
11. López N, Cuesta G, Rodríguez-Vega S, Rosas E, Chumbita M, Casals-Pascual C, Morata L, Vergara A, Bodro M, Bosch J, Herrera S, Martínez JA, Mensa J, Garcia-Vidal C, Marcos MÁ, Vila J, Soriano A, Puerta-Alcalde P. Multiplex real-time PCR FilmArray performance in the diagnosis of meningoencephalitis: lights and shadows. *Infection.* 2024 Feb;52(1):165-172. doi: 10.1007/s15010-023-02076-x. Epub 2023 Jul 29. PMID: 37515691; PMCID: PMC10810907.
12. *Boers SA, van Houdt R, van Sorge NM, Groot J, van Aarle Y, van Bussel MJAWM, Smit LFE, Wessels E, Claas ECJ. A multicenter evaluation of the QIAstat-Dx meningitis-encephalitis syndromic test kit as compared to the conventional diagnostic microbiology workflow. *Eur J Clin Microbiol Infect Dis.* 2024 Mar;43(3):511-516. doi: 10.1007/s10096-024-04751-9. Epub 2024 Jan 11. PMID: 38206519; PMCID: PMC10917839.
13. Obaro S, Hassan-Hanga F, Medugu N, Olaosebikan R, Olanipekun G, Jibir B, Gambo S, Ajose T, Duru C, Ebruke B, Davies HD. Comparison of bacterial culture with BioFire® FilmArray® multiplex PCR screening of archived cerebrospinal fluid specimens from children with suspected bacterial meningitis in Nigeria. *BMC Infect Dis.* 2023 Oct 2;23(1):641. doi: 10.1186/s12879-023-08645-7. PMID: 37784010; PMCID: PMC10544496.
14. Ghoweba Y, Safizadeh Shabestari SA, Malik ZA. Diagnostic Accuracy of Cerebrospinal Fluid Multiplex Polymerase Chain Reaction Panel Testing in Patients With Suspected Central Nervous System Infections: A Multi-Center Study in the United Arab Emirates. *Cureus.* 2024 Jan 8;16(1):e51906. doi: 10.7759/cureus.51906. PMID: 38333447; PMCID: PMC10851033.

### Metagenomics

1. **Benoit P, Brazer N, de Lorenzi-Tognon M, Kelly E, Servellita V, Oseguera M, Nguyen J, Tang J, Omura C, Streithorst J, Hillberg M, Ingebrigtsen D, Zorn K, Wilson MR, Blicharz T, Wong AP, O'Donovan B, Murray B, Miller S, Chiu CY. Seven-year performance of a clinical metagenomic next-generation sequencing test for diagnosis of central nervous system infections. Nat Med. 2024 Dec;30(12):3522-3533. doi: 10.1038/s41591-024-03275-1. Epub 2024 Nov 12. PMID: 39533109; PMCID: PMC11645279.
2. **Zou YS, Cao ZL, Guo Y, Wang BB, Wang JL, Cheng R, Yang Y, Zhou XG. The application value of metagenomic next-generation sequencing technology in the diagnosis and treatment of neonatal infectious meningitis - a single center retrospective case-control study. Neurol Res. 2024 Jun;46(6):561-567. doi: 10.1080/01616412.2024.2337523. Epub 2024 Apr 2. PMID: 38563313.
3. *Chiu CY, Godasi RR, Hughes HR, Servellita V, Foresythe K, Tubati A, et al. Two Human Cases of Fatal Meningoencephalitis Associated with Potosi and Lone Star Virus Infections, United States, 2020-2023. Emerg Infect Dis. 2025;31(2):215-21.
4. *He S, Xiong Y, Tu T, Feng J, Fu Y, Hu X, Wang N, Li D. Diagnostic performance of metagenomic next-generation sequencing for the detection of pathogens in cerebrospinal fluid in pediatric patients with central nervous system infection: a systematic review and meta-analysis. BMC Infect Dis. 2024 Jan 18;24(1):103. doi: 10.1186/s12879-024-09010-y. PMID: 38238719; PMCID: PMC10797782.
5. *Wang X, Guo X, Liu H, Wang B, Wu J, Chen S, Zhang W, Zhang X, Wang X. Augmented pathogen detection in brain abscess using metagenomic next-generation sequencing: a retrospective cohort study. Microbiol Spectr. 2024 Oct 3;12(10):e0032524. doi: 10.1128/spectrum.00325-24. Epub 2024 Sep 12. PMID: 39264158; PMCID: PMC11448231.
6. *Gámbaro F, Pérez AB, Prot M, Agüera E, Baidaliuk A, Sánchez-Seco MP, Martínez-Martínez L, Vázquez A, Fernandez-Garcia MD, Simon-Loriere E. Untargeted metagenomic sequencing identifies Toscana virus in patients with idiopathic meningitis, southern Spain, 2015 to 2019. Euro Surveill. 2023 Nov;28(45):2200913. doi: 10.2807/1560-7917.ES.2023.28.45.2200913. PMID: 37943504; PMCID: PMC10636744.
7. *Castellot A, Camacho J, Fernández-García MD, Tarragó D. Shotgun metagenomics to investigate unknown viral etiologies of pediatric meningoencephalitis. PLoS One. 2023 Dec 21;18(12):e0296036. doi: 10.1371/journal.pone.0296036. PMID: 38127927; PMCID: PMC10734945.
8. *Launes C, Camacho J, Pons-Espinal M, López-Labrador FX, Esteva C, Cabrerizo M, Fernández-García MD, Fogeda M, Masa-Calles J, López-Perea N, Echevarría JE, Muñoz-Almagro C, Tarragó D. Hybrid capture shotgun sequencing detected unexpected viruses in the cerebrospinal fluid of children with acute meningitis and encephalitis. Eur J Clin Microbiol Infect Dis. 2024 May;43(5):863-873. doi: 10.1007/s10096-024-04795-x. Epub 2024 Mar 4. PMID: 38438704; PMCID: PMC11108891.
9. Zhang K, Xu J, Chen G, Yang R, Jiang M, Yuan H. Metagenomic Next-Generation Sequencing (mNGS) of cerebrospinal fluid for diagnosis of human herpesvirus 6B encephalitis following transplantation for severe aplastic anemia. J Infect Dev Ctries. 2024;18(1):152-7.
10. *Zou S, Chen Z, Tan Y, Tan M, Guo W, Wu S, et al. Microbiomes detected by cerebrospinal fluid metagenomic next-generation sequencing among patients with and without HIV with suspected central nervous system infection. HIV Med. 2024;25(7):794-804.
11. *Smith DJ, Gold JAW, Chiller T, Bustamante ND, Marinissen MJ, Rodriquez GG, Cortes VBG, Molina CD, Williams S, Vazquez Deida AA, Byrd K, Pappas PG, Patterson TF, Wiederhold NP, Thompson GR 3rd, Ostrosky-Zeichner L; Fungal Meningitis Response Team. Update on Outbreak of Fungal Meningitis Among US Residents Who Received Epidural Anesthesia at Two Clinics in Matamoros, Mexico. Clin Infect Dis. 2024 Jun 14;78(6):1554-1558. doi: 10.1093/cid/ciad570. PMID: 37739479; PMCID: PMC10957502.
12. Fourgeaud J, Regnault B, Ok V, Da Rocha N, Sitterlé É, Mekouar M, Faury H, Milliancourt-Seels C, Jagorel F, Chrétien D, Bigot T, Troadec É, Marques I, Serris A, Seilhean D, Neven B, Frange P, Ferroni A, Lecuit M, Nassif X, Lortholary O, Leruez-Ville M, Pérot P, Eloit M, Jamet A. Performance of clinical metagenomics in France: a prospective observational study. Lancet Microbe. 2024 Jan;5(1):e52-e61. doi: 10.1016/S2666-5247(23)00244-6. Epub 2023 Dec 1. PMID: 38048804.
13. Shean RC, Garrett E, Malleis J, Lieberman JA, Bradley BT. A retrospective observational study of mNGS test utilization to examine the role of diagnostic stewardship at two academic medical centers. J Clin Microbiol. 2024 Sep 11;62(9):e0060524. doi: 10.1128/jcm.00605-24. Epub 2024 Aug 20. PMID: 39162437; PMCID: PMC11389146.
14. Steinberg HE, Ramachandran PS, Diestra A, Pinchi L, Ferradas C, Kirwan DE, Diaz MM, Sciaudone M, Wapniarski A, Zorn KC, Calderón M, Cabrera L, Pinedo-Cancino V, Wilson MR, Asayag CR, Gilman RH, Bowman NM; Toxoplasmosis Working Group in Peru. Clinical and Metagenomic Characterization of Neurological Infections of People With Human Immunodeficiency Virus in the Peruvian Amazon. Open Forum Infect Dis. 2023 Oct 27;10(11):ofad515. doi: 10.1093/ofid/ofad515. PMID: 37965640; PMCID: PMC10642733.

### Pathogen-based protein and metabolite biomarkers “antigens”

1. *Lin HL, Chen HM, Lin CY, Chen CC. Accuracy of antigen tests for meningococcal meningitis in cerebrospinal fluid: A diagnostic meta-analysis. *Trop Med Int Health.* 2023 Oct;28(10):797-805. doi: 10.1111/tmi.13928. Epub 2023 Aug 28. PMID: 37641441.
2. *Bigot J, Leroy J, Chouaki T, Cholley L, Bigé N, Tabone MD, Brissot E, Thorez S, Maizel J, Dupont H, Sendid B, Hennequin C, Guitard J. ß-D-Glucan Assay in the Cerebrospinal Fluid for the Diagnosis of Non-cryptococcal Fungal Infection of the Central Nervous System: A Retrospective Multicentric Analysis and a Comprehensive Review of the Literature. Clin Infect Dis. 2023 Sep 11;77(5):711-720. doi: 10.1093/cid/ciad274. PMID: 37132362.
3. *Komorowski AS, Hall CW, Atwal S, Johnstone R, Walker R 3rd, Mertz D, Piessens EA, Yamamura D, Kasper EM. Cerebrospinal fluid galactomannan detection for the diagnosis of central nervous system aspergillosis: a diagnostic test accuracy systematic review and meta-analysis. Clin Microbiol Infect. 2024 Oct;30(10):1244-1253. doi: 10.1016/j.cmi.2024.05.013. Epub 2024 May 27. PMID: 38810927.
4. Gupta-Wright A, Ha H, Abdulgadar S, Crowder R, Emmanuel J, Mukwatamundu J, et al. Evaluation of the Xpert MTB Host Response assay for the triage of patients with presumed pulmonary tuberculosis: a prospective diagnostic accuracy study in Viet Nam, India, the Philippines, Uganda, and South Africa. The Lancet Global Health. 2024;12(2):e226-e34.

### Intrathecal antibodies

1. *Li FF, Faber A, Caleta JM, Goldfarb DM, Sekirov I, Prystajecky NA, Srigley JA, Mishaal R, Jassem AN. Clinical application of phage immunoprecipitation sequencing to diagnose enterovirus D68 as the underlying etiology in a case of Gullain-Barré syndrome. J Infect Dis. 2024 Aug 31:jiae411. doi: 10.1093/infdis/jiae411. Epub ahead of print. PMID: 39215587
2. *Platz IL, Tetens MM, Dessau R, Ellermann-Eriksen S, Andersen NS, Jensen VVS, Østergaard C, Bodilsen J, Søgaard KK, Bangsborg J, Nielsen ACY, Møller JK, Lebech AM, Omland LH, Obel N. Characteristics and long-term prognosis of Danish residents with a positive intrathecal antibody index test for herpes simplex virus or varicella-zoster virus compared with individuals with a positive cerebrospinal fluid PCR: a nationwide cohort study. Clin Microbiol Infect. 2024 Feb;30(2):240-246. doi: 10.1016/j.cmi.2023.11.004. Epub 2023 Nov 13. PMID: 37967615.
3. *Garcia R, Jiménez-Valera M, Ruiz-Buck D, Sanchez C, Rojas A, Schütz MH, Rojas J, Hunfeld KP. Detection of intrathecal IgG antibody for varicella and measles diagnosis by evaluation and comparison of a commercial IgG chemiluminescent immunoassay with two ELISAs. Eur J Clin Microbiol Infect Dis. 2024 Jun;43(6):1139-1148. doi: 10.1007/s10096-024-04822-x. Epub 2024 Apr 13. PMID: 38613705.
4. *Lee CY, Liao CW, Wang LC, Fan CK, Chuang TW, En-Te Hwu E, Chao D, Cheng PC. IgE antibody responses in cerebrospinal fluids relate to the brain pathologic injury of hosts with Angiostrongylus cantonensis infection. J Microbiol Immunol Infect. 2023 Dec;56(6):1261-1272. doi: 10.1016/j.jmii.2023.08.012. Epub 2023 Sep 4. PMID: 37689501
5. *Czupryna P, Grygorczuk S, Siemieniako-Werszko A, Okrzeja J, Dunaj-Małyszko J, Adamczuk J, Pancewicz S, Zajkowska J, Narejko K, Oklińska J, Trojan G, Moniuszko-Malinowska A. Anti-Tick-Bourne Encephalitis IgM Intrathecal Synthesis as a Prediction Marker in Tick-Borne Encephalitis Patients. Microorganisms. 2025 Jan 20;13(1):213. doi: 10.3390/microorganisms13010213. PMID: 39858981; PMCID: PMC11767730

### Transcriptomics and proteomics

1. **Huynh J, Nhat LHT, Bao NLH, Hai HT, Thu DDA, Tram TTB, Dung VTM, Vinh DD, Ngoc NM, Donovan J, Phu NH, Van Thanh D, Thu NTA, Bang ND, Ha DTM, Nghia HDT, Van Tan L, Van LH, Thwaites G, Thuong NTT. The Ability of a 3-Gene Host Signature in Blood to Distinguish Tuberculous Meningitis From Other Brain Infections. J Infect Dis. 2024 Aug 16;230(2):e268-e278. doi: 10.1093/infdis/jiad606. PMID: 38169323; PMCID: PMC11326836.
2. **Groeneveld NS, Olie SE, Visser DH, Snoek L, van de Beek D, Brouwer MC, Bijlsma MW; NOGBS study group. Cerebrospinal fluid inflammatory markers to differentiate between neonatal bacterial meningitis and sepsis: A prospective study of diagnostic accuracy. Int J Infect Dis. 2024 May;142:106970. doi: 10.1016/j.ijid.2024.02.013. Epub 2024 Feb 21. PMID: 38395221.
3. *Zachariassen M, Thomsen MM, Hillig T, Trier-Petersen P, Jensen AV, Friis-Hansen LJ, Brandt CT. Tenascin-C in patients with central nervous system infections. J Neuroimmunol. 2024 Jul 15;392:578373. doi: 10.1016/j.jneuroim.2024.578373. Epub 2024 May 15. PMID: 38776710.
4. *Olie SE, Staal SL, da Cruz Campos AC, Bodilsen J, Nielsen H, van de Beek D, Brouwer MC. Heparin-Binding Protein in Cerebrospinal Fluid as a Biomarker for Bacterial Meningitis: A Study of Diagnostic Accuracy. Ann Neurol. 2025 Jan 27. doi: 10.1002/ana.27193. Epub ahead of print. PMID: 39868663.
5. *Biasucci DG, Sergi PG, Bilotta F, Dauri M. Diagnostic Accuracy of Procalcitonin in Bacterial Infections of the CNS: An Updated Systematic Review, Meta-Analysis, and Meta-Regression. Crit Care Med. 2024;52(1):112-24.
6. *Alsén K, Patzi Churqui M, Norder H, Rembeck K, Zetterberg H, Blennow K, et al. Biomarkers and genotypes in patients with Central nervous system infection caused by enterovirus. Infect Dis (Lond). 2024;56(9):722-31.
7. Olie SE, Staal SL, Ter Horst L, van Zeggeren IE, Man WK, Tanck MWT, van de Beek D, Brouwer MC. Diagnostic accuracy of inflammatory markers in adults with suspected central nervous system infections. J Infect. 2024 Mar;88(3):106117. doi: 10.1016/j.jinf.2024.01.016. Epub 2024 Feb 5. PMID: 38320644; PMCID: PMC10943182.
8. Baran A, Huyut Z, Öncü MR, Akbay H, Akmeşe Ş, Karsen H, et al. Evaluation of cerebrospinal fluid levels for ALOX5, S100B, DEFA1, and GFAP in infectious meningitis. Medicine (Baltimore). 2023;102(50):e36463.
9. Yao X-P, Hong J-C, Jiang Z-J, Pan Y-Y, Liu X-F, Wang J-M, Fan R-J, Yang B-H, Zhang W-Q, Fan Q-C, Li L-X, Lin B-W, Zhao M. Systemic and cerebrospinal fluid biomarkers for tuberculous meningitis identification and treatment monitoring. Microbiol Spectr. 2024 Jan 11;12(1):e0224623. doi: 10.1128/spectrum.02246-23. Epub 2023 Dec 4. PMID: 38047697; PMCID: PMC10783035.
10. Damodar T, Dunai C, Prabhu N, Jose M, Akhila L, Kinhal UV, Anusha Raj K, Marate S, Lalitha AV, Dsouza FS, Sajjan SV, Gowda VK, Basavaraja GV, Singh B, Prathyusha PV, Tharmaratnam K, Ravi V, Kolamunnage-Dona R, Solomon T, Turtle L, Yadav R, Michael BD, Mani RS. Diagnostic markers of acute encephalitis syndrome and COVID-associated multisystem inflammatory syndrome in children from Southern India. J Med Virol. 2024 May;96(5):e29666. doi: 10.1002/jmv.29666. PMID: 38738569; PMCID: PMC7616670
11. Liang R, Li Y, Li J, Zhang S, Gao Y, Tan F, Feng Y, Chen Y, Wang F, Jiang T, Kang X. Metabolomic profiling of cerebrospinal fluid reveals metabolite biomarkers in tick-borne encephalitis patient. J Med Virol. 2024 Nov;96(11):e70082. doi: 10.1002/jmv.70082. PMID: 39569456; PMCID: PMC11579828.
12. Wang Q, Lin Q, Wang H, Tang M, Fan K, Zhang Z, Huang E, Zhang W, Wang F, Ou Q, Liu X. Diagnostic value of cerebrospinal fluid Neutrophil Gelatinase-Associated Lipocalin for differentiation of bacterial meningitis from tuberculous meningitis or cryptococcal meningitis: a prospective cohort study. J Transl Med. 2023 Sep 7;21(1):603. doi: 10.1186/s12967-023-04485-w. PMID: 37679727; PMCID: PMC10486126.

### Metabolomics

1. *Al-Mekhlafi A, Waqas FH, Krueger M, Klawonn F, Akmatov MK, Müller-Vahl K, Trebst C, Skripuletz T, Stangel M, Sühs KW, Pessler F. Elevated phospholipids and acylcarnitines C4 and C5 in cerebrospinal fluid distinguish viral CNS infections from autoimmune neuroinflammation. J Transl Med. 2023 Nov 2;21(1):776. doi: 10.1186/s12967-023-04637-y. PMID: 37919735; PMCID: PMC10621113.
2. *Chen YL, Zhu MM, Guan CP, Zhang YA, Wang MS. Diagnostic value of the cerebrospinal fluid lipoarabinomannan assay for tuberculous meningitis: a systematic review and meta-analysis. Front Public Health. 2023 Sep 21;11:1228134. doi: 10.3389/fpubh.2023.1228134. PMID: 37808998; PMCID: PMC10552265.
